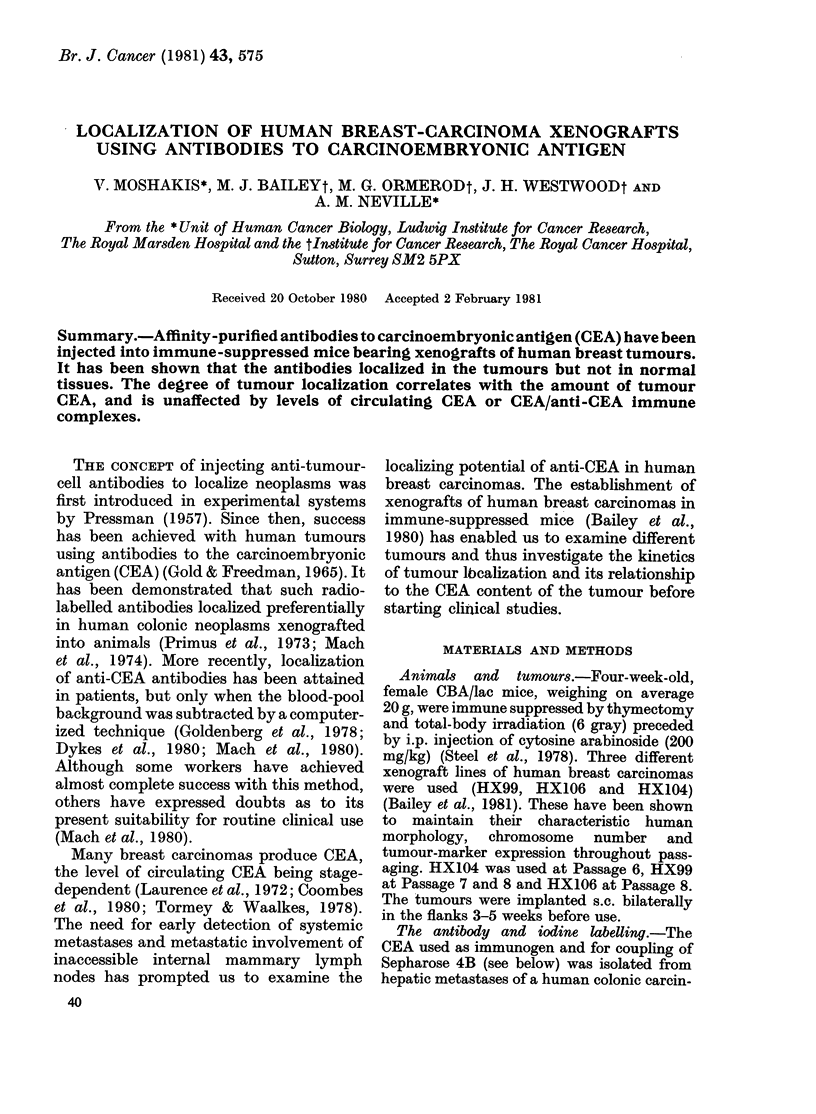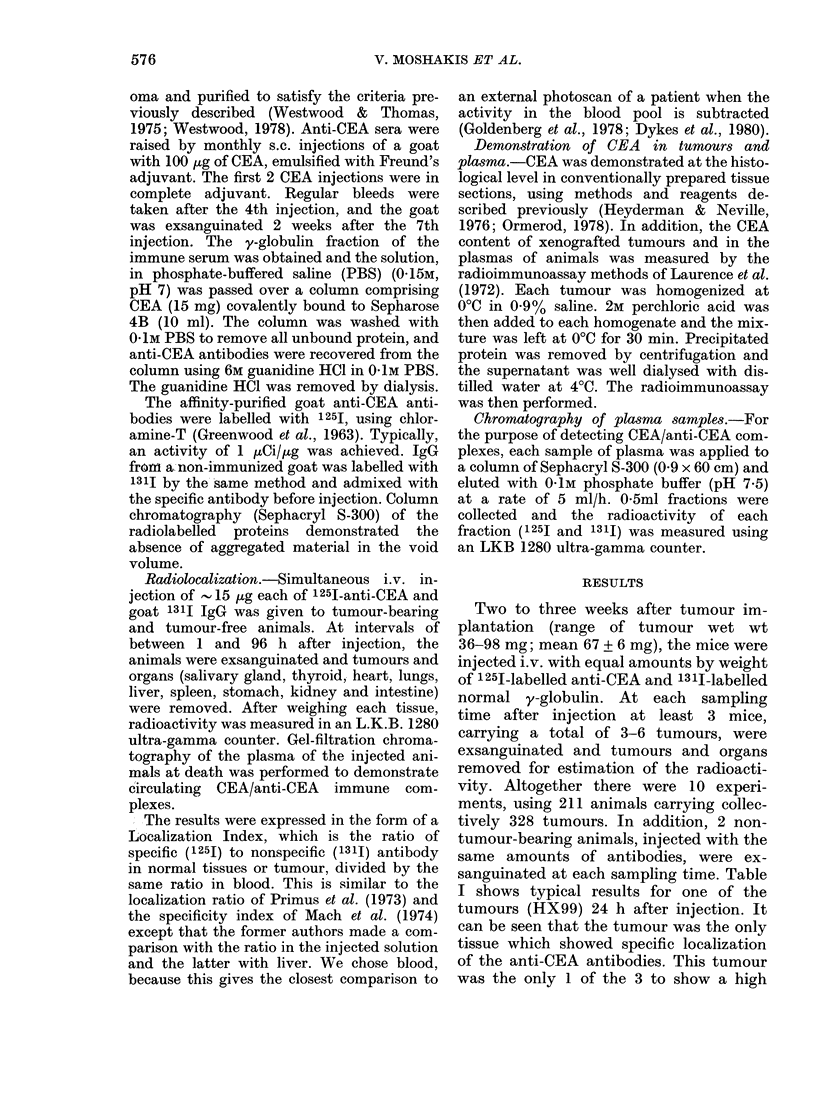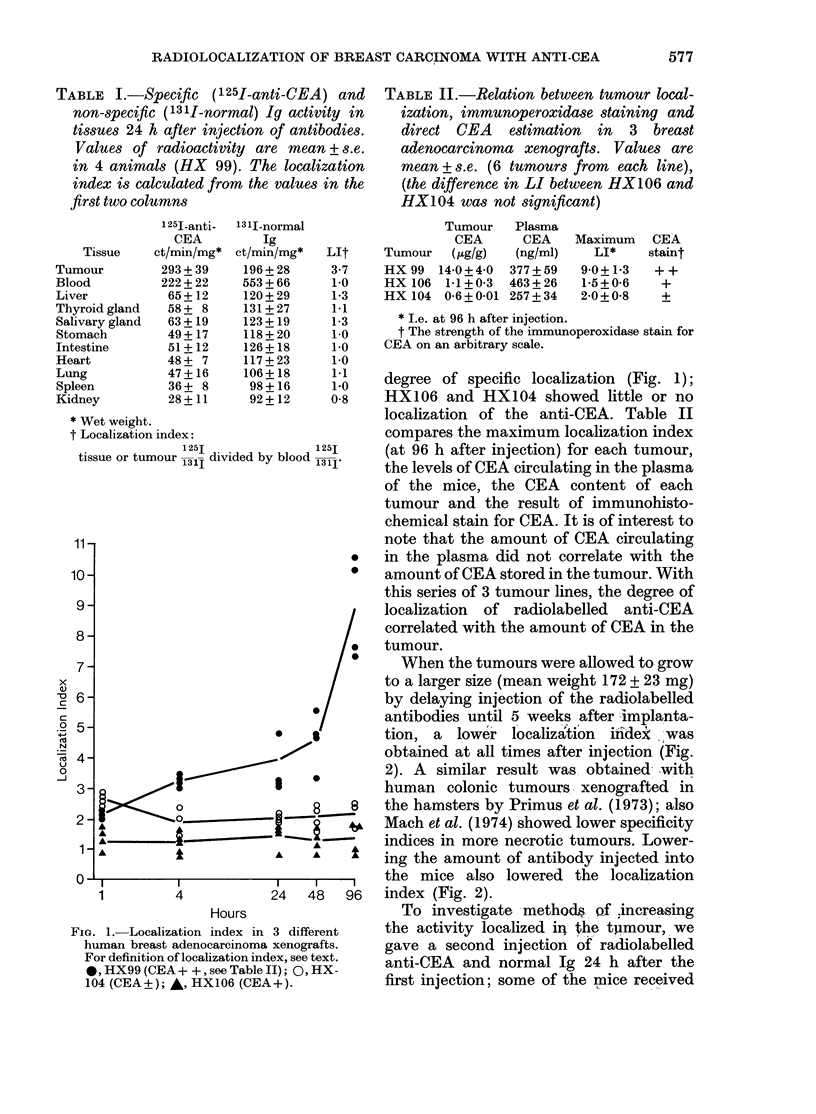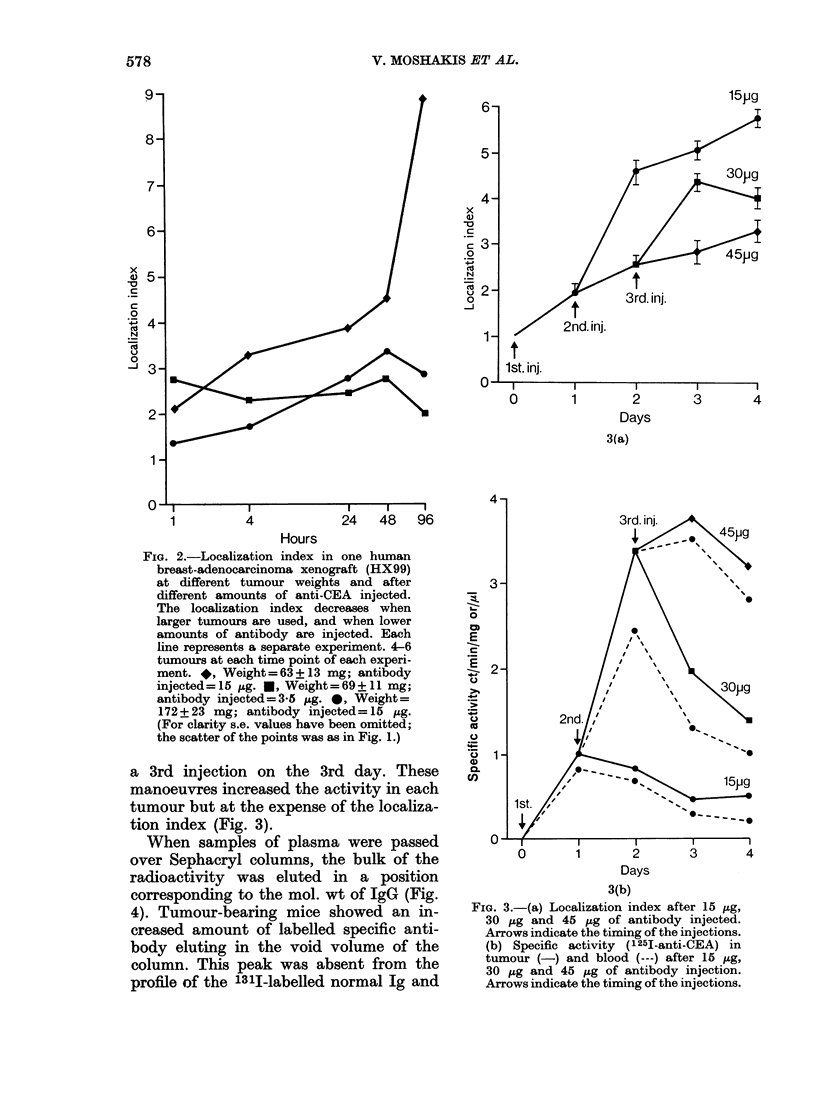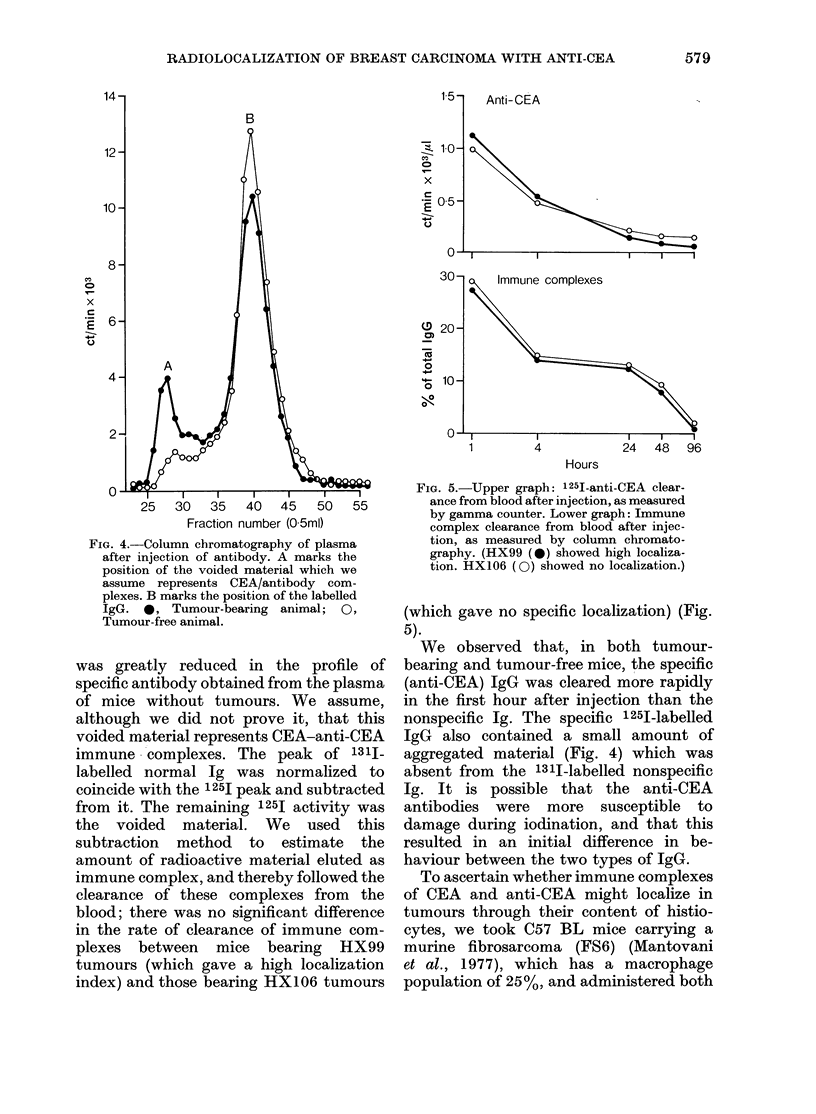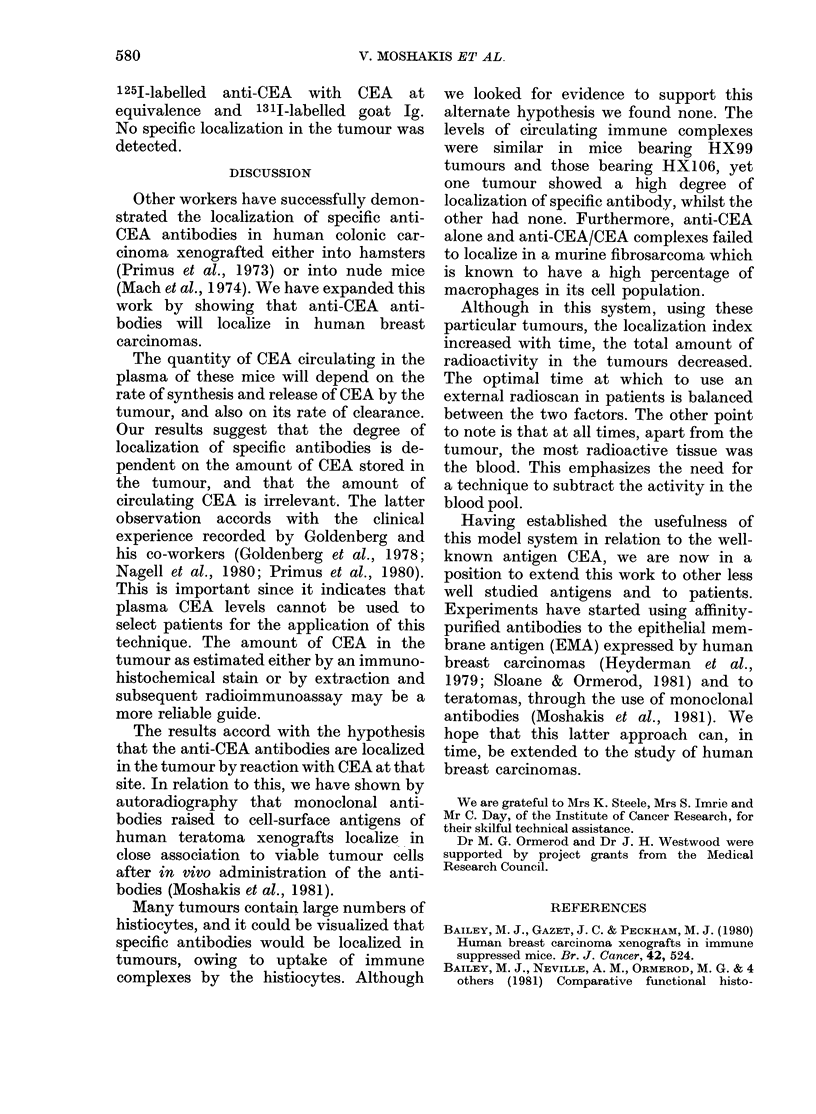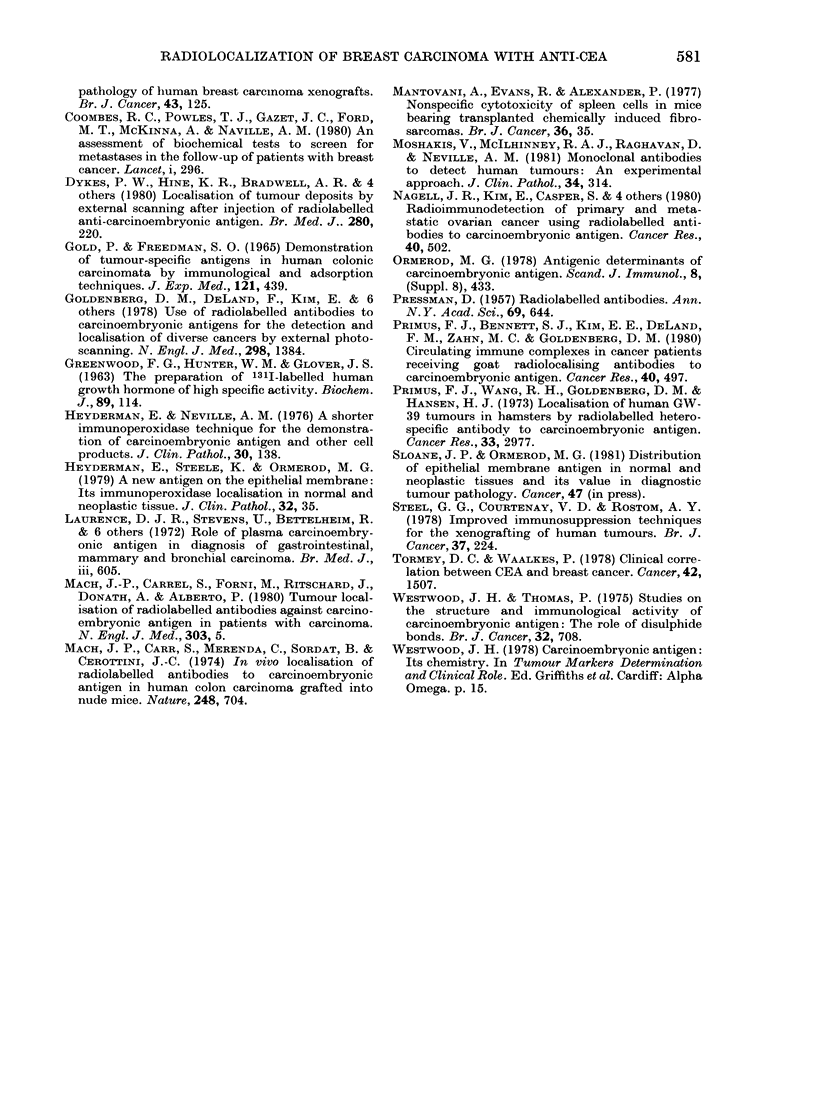# Localization of human breast-carcinoma xenografts using antibodies to carcinoembryonic antigen.

**DOI:** 10.1038/bjc.1981.86

**Published:** 1981-05

**Authors:** V. Moshakis, M. J. Bailey, M. G. Ormerod, J. H. Westwood, A. M. Neville

## Abstract

Affinity-purified antibodies to carcinoembryonic antigen (CEA) have been injected into immune-suppressed mice bearing xenografts of human breast tumours. It has been shown that the antibodies localized in the tumours but not in normal tissues. The degree of tumour localization correlates with the amount of tumour CEA, and is unaffected by levels of circulating CEA or CEA/anti-CEA immune complexes.


Br. J. Cancer (1981) 43, 575

### LOCALIZATION OF HUMAN BREAST-CARCINOMA XENOGRAFTS USING ANTIBODIES TO CARCINOEMBRYONIC ANTIGEN

V. MOSHAKIS*, M. J. BAILEY†, M. G. ORMEROD†, J. H. WESTWOOD† AND A. M. NEVILLE*

*From the *Unit of Human Cancer Biology, Ludwig Institute for Cancer Research, The Royal Marsden Hospital and the †Institute for Cancer Research, The Royal Cancer Hospital, Sutton, Surrey SM2 5PX*

Received 20 October 1980 Accepted 2 February 1981

**Summary.—Affinity-purified antibodies to carcinoembryonic antigen (CEA) have been injected into immune-suppressed mice bearing xenografts of human breast tumours. It has been shown that the antibodies localized in the tumours but not in normal tissues. The degree of tumour localization correlates with the amount of tumour CEA, and is unaffected by levels of circulating CEA or CEA/anti-CEA immune complexes.**

THE CONCEPT of injecting anti-tumour-cell antibodies to localize neoplasms was first introduced in experimental systems by Pressman (1957). Since then, success has been achieved with human tumours using antibodies to the carcinoembryonic antigen (CEA) (Gold & Freedman, 1965). It has been demonstrated that such radio-labelled antibodies localized preferentially in human colonic neoplasms xenografted into animals (Primus *et al.*, 1973; Mach *et al.*, 1974). More recently, localization of anti-CEA antibodies has been attained in patients, but only when the blood-pool background was subtracted by a computerized technique (Goldenberg *et al.*, 1978; Dykes *et al.*, 1980; Mach *et al.*, 1980). Although some workers have achieved almost complete success with this method, others have expressed doubts as to its present suitability for routine clinical use (Mach *et al.*, 1980).

Many breast carcinomas produce CEA, the level of circulating CEA being stage-dependent (Laurence *et al.*, 1972; Coombes *et al.*, 1980; Tormey & Waalkes, 1978). The need for early detection of systemic metastases and metastatic involvement of inaccessible internal mammary lymph nodes has prompted us to examine the localizing potential of anti-CEA in human breast carcinomas. The establishment of xenografts of human breast carcinomas in immune-suppressed mice (Bailey *et al.*, 1980) has enabled us to examine different tumours and thus investigate the kinetics of tumour localization and its relationship to the CEA content of the tumour before starting clinical studies.

### MATERIALS AND METHODS

*Animals and tumours.*—Four-week-old, female CBA/lac mice, weighing on average 20 g, were immune suppressed by thymectomy and total-body irradiation (6 gray) preceded by i.p. injection of cytosine arabinoside (200 mg/kg) (Steel *et al.*, 1978). Three different xenograft lines of human breast carcinomas were used (HX99, HX106 and HX104) (Bailey *et al.*, 1981). These have been shown to maintain their characteristic human morphology, chromosome number and tumour-marker expression throughout passaging. HX104 was used at Passage 6, HX99 at Passage 7 and 8 and HX106 at Passage 8. The tumours were implanted s.c. bilaterally in the flanks 3–5 weeks before use.

*The antibody and iodine labelling.*—The CEA used as immunogen and for coupling of Sepharose 4B (see below) was isolated from hepatic metastases of a human colonic carcinoma and purified to satisfy the criteria previously described (Westwood & Thomas, 1975; Westwood, 1978). Anti-CEA sera were raised by monthly s.c. injections of a goat with 100 $\mu$g of CEA, emulsified with Freund's adjuvant. The first 2 CEA injections were in complete adjuvant. Regular bleeds were taken after the 4th injection, and the goat was exsanguinated 2 weeks after the 7th injection. The $\gamma$-globulin fraction of the immune serum was obtained and the solution, in phosphate-buffered saline (PBS) (0·15M, pH 7) was passed over a column comprising CEA (15 mg) covalently bound to Sepharose 4B (10 ml). The column was washed with 0·1M PBS to remove all unbound protein, and anti-CEA antibodies were recovered from the column using 6M guanidine HCl in 0·1M PBS. The guanidine HCl was removed by dialysis.

The affinity-purified goat anti-CEA antibodies were labelled with $^{125}$I, using chloramine-T (Greenwood *et al.*, 1963). Typically, an activity of 1 $\mu$Ci/$\mu$g was achieved. IgG from a non-immunized goat was labelled with $^{131}$I by the same method and admixed with the specific antibody before injection. Column chromatography (Sephacryl S-300) of the radiolabelled proteins demonstrated the absence of aggregated material in the void volume.

*Radiolocalization.*—Simultaneous i.v. injection of $\sim$15 $\mu$g each of $^{125}$I-anti-CEA and goat $^{131}$I IgG was given to tumour-bearing and tumour-free animals. At intervals of between 1 and 96 h after injection, the animals were exsanguinated and tumours and organs (salivary gland, thyroid, heart, lungs, liver, spleen, stomach, kidney and intestine) were removed. After weighing each tissue, radioactivity was measured in an L.K.B. 1280 ultra-gamma counter. Gel-filtration chromatography of the plasma of the injected animals at death was performed to demonstrate circulating CEA/anti-CEA immune complexes.

The results were expressed in the form of a Localization Index, which is the ratio of specific ($^{125}$I) to nonspecific ($^{131}$I) antibody in normal tissues or tumour, divided by the same ratio in blood. This is similar to the localization ratio of Primus *et al.* (1973) and the specificity index of Mach *et al.* (1974) except that the former authors made a comparison with the ratio in the injected solution and the latter with liver. We chose blood, because this gives the closest comparison to an external photoscan of a patient when the activity in the blood pool is subtracted (Goldenberg *et al.*, 1978; Dykes *et al.*, 1980).

*Demonstration of CEA in tumours and plasma.*—CEA was demonstrated at the histological level in conventionally prepared tissue sections, using methods and reagents described previously (Heyderman & Neville, 1976; Ormerod, 1978). In addition, the CEA content of xenografted tumours and in the plasmas of animals was measured by the radioimmunoassay methods of Laurence *et al.* (1972). Each tumour was homogenized at 0°C in 0·9% saline. 2M perchloric acid was then added to each homogenate and the mixture was left at 0°C for 30 min. Precipitated protein was removed by centrifugation and the supernatant was well dialysed with distilled water at 4°C. The radioimmunoassay was then performed.

*Chromatography of plasma samples.*—For the purpose of detecting CEA/anti-CEA complexes, each sample of plasma was applied to a column of Sephacryl S-300 (0·9 × 60 cm) and eluted with 0·1M phosphate buffer (pH 7·5) at a rate of 5 ml/h. 0·5ml fractions were collected and the radioactivity of each fraction ($^{125}$I and $^{131}$I) was measured using an LKB 1280 ultra-gamma counter.

### RESULTS

Two to three weeks after tumour implantation (range of tumour wet wt 36–98 mg; mean 67 ± 6 mg), the mice were injected i.v. with equal amounts by weight of $^{125}$I-labelled anti-CEA and $^{131}$I-labelled normal $\gamma$-globulin. At each sampling time after injection at least 3 mice, carrying a total of 3–6 tumours, were exsanguinated and tumours and organs removed for estimation of the radioactivity. Altogether there were 10 experiments, using 211 animals carrying collectively 328 tumours. In addition, 2 non-tumour-bearing animals, injected with the same amounts of antibodies, were exsanguinated at each sampling time. Table I shows typical results for one of the tumours (HX99) 24 h after injection. It can be seen that the tumour was the only tissue which showed specific localization of the anti-CEA antibodies. This tumour was the only 1 of the 3 to show a high

TABLE I.—*Specific* ($^{125}$*I-anti-CEA*) *and non-specific* ($^{131}$*I-normal*) *Ig activity in tissues 24 h after injection of antibodies. Values of radioactivity are mean* ± *s.e. in 4 animals* (*HX* 99). *The localization index is calculated from the values in the first two columns*

| Tissue | $^{125}$I-anti-CEA ct/min/mg* | $^{131}$I-normal Ig ct/min/mg* | LI† |
|---|---|---|---|
| Tumour | 293 ± 39 | 196 ± 28 | 3·7 |
| Blood | 222 ± 22 | 553 ± 66 | 1·0 |
| Liver | 65 ± 12 | 120 ± 29 | 1·3 |
| Thyroid gland | 58 ± 8 | 131 ± 27 | 1·1 |
| Salivary gland | 63 ± 19 | 123 ± 19 | 1·3 |
| Stomach | 49 ± 17 | 118 ± 20 | 1·0 |
| Intestine | 51 ± 12 | 126 ± 18 | 1·0 |
| Heart | 48 ± 7 | 117 ± 23 | 1·0 |
| Lung | 47 ± 16 | 106 ± 18 | 1·1 |
| Spleen | 36 ± 8 | 98 ± 16 | 1·0 |
| Kidney | 28 ± 11 | 92 ± 12 | 0·8 |

* Wet weight.
† Localization index:

tissue or tumour $\frac{^{125}I}{^{131}I}$ divided by blood $\frac{^{125}I}{^{131}I}$.

FIG. 1.—Localization index in 3 different human breast adenocarcinoma xenografts. For definition of localization index, see text. ●, HX99 (CEA + +, see Table II); ○, HX-104 (CEA ±); ▲, HX106 (CEA +).

TABLE II.—*Relation between tumour localization, immunoperoxidase staining and direct CEA estimation in 3 breast adenocarcinoma xenografts. Values are mean* ± *s.e.* (6 *tumours from each line*), (*the difference in LI between HX*106 *and HX*104 *was not significant*)

| Tumour | Tumour CEA (μg/g) | Plasma CEA (ng/ml) | Maximum LI* | CEA stain† |
|---|---|---|---|---|
| HX 99 | 14·0 ± 4·0 | 377 ± 59 | 9·0 ± 1·3 | + + |
| HX 106 | 1·1 ± 0·3 | 463 ± 26 | 1·5 ± 0·6 | + |
| HX 104 | 0·6 ± 0·01 | 257 ± 34 | 2·0 ± 0·8 | ± |

* I.e. at 96 h after injection.
† The strength of the immunoperoxidase stain for CEA on an arbitrary scale.

degree of specific localization (Fig. 1); HX106 and HX104 showed little or no localization of the anti-CEA. Table II compares the maximum localization index (at 96 h after injection) for each tumour, the levels of CEA circulating in the plasma of the mice, the CEA content of each tumour and the result of immunohistochemical stain for CEA. It is of interest to note that the amount of CEA circulating in the plasma did not correlate with the amount of CEA stored in the tumour. With this series of 3 tumour lines, the degree of localization of radiolabelled anti-CEA correlated with the amount of CEA in the tumour.

When the tumours were allowed to grow to a larger size (mean weight 172 ± 23 mg) by delaying injection of the radiolabelled antibodies until 5 weeks after implantation, a lower localization index was obtained at all times after injection (Fig. 2). A similar result was obtained with human colonic tumours xenografted in the hamsters by Primus *et al.* (1973); also Mach *et al.* (1974) showed lower specificity indices in more necrotic tumours. Lowering the amount of antibody injected into the mice also lowered the localization index (Fig. 2).

To investigate methods of increasing the activity localized in the tumour, we gave a second injection of radiolabelled anti-CEA and normal Ig 24 h after the first injection; some of the mice received

FIG. 2.—Localization index in one human breast-adenocarcinoma xenograft (HX99) at different tumour weights and after different amounts of anti-CEA injected. The localization index decreases when larger tumours are used, and when lower amounts of antibody are injected. Each line represents a separate experiment. 4–6 tumours at each time point of each experiment. ◆, Weight $= 63 \pm 13$ mg; antibody injected $= 15$ $\mu$g. ■, Weight $= 69 \pm 11$ mg; antibody injected $= 3{\cdot}5$ $\mu$g. ●, Weight $= 172 \pm 23$ mg; antibody injected $= 15$ $\mu$g. (For clarity s.e. values have been omitted; the scatter of the points was as in Fig. 1.)

a 3rd injection on the 3rd day. These manoeuvres increased the activity in each tumour but at the expense of the localization index (Fig. 3).

When samples of plasma were passed over Sephacryl columns, the bulk of the radioactivity was eluted in a position corresponding to the mol. wt of IgG (Fig. 4). Tumour-bearing mice showed an increased amount of labelled specific antibody eluting in the void volume of the column. This peak was absent from the profile of the $^{131}$I-labelled normal Ig and

3(a)

3(b)

FIG. 3.—(a) Localization index after 15 $\mu$g, 30 $\mu$g and 45 $\mu$g of antibody injected. Arrows indicate the timing of the injections. (b) Specific activity ($^{125}$I-anti-CEA) in tumour (—) and blood (---) after 15 $\mu$g, 30 $\mu$g and 45 $\mu$g of antibody injection. Arrows indicate the timing of the injections.

FIG. 4.—Column chromatography of plasma after injection of antibody. A marks the position of the voided material which we assume represents CEA/antibody complexes. B marks the position of the labelled IgG. ●, Tumour-bearing animal; ○, Tumour-free animal.

FIG. 5.—Upper graph: $^{125}$I-anti-CEA clearance from blood after injection, as measured by gamma counter. Lower graph: Immune complex clearance from blood after injection, as measured by column chromatography. (HX99 (●) showed high localization. HX106 (○) showed no localization.)

was greatly reduced in the profile of specific antibody obtained from the plasma of mice without tumours. We assume, although we did not prove it, that this voided material represents CEA–anti-CEA immune complexes. The peak of $^{131}$I-labelled normal Ig was normalized to coincide with the $^{125}$I peak and subtracted from it. The remaining $^{125}$I activity was the voided material. We used this subtraction method to estimate the amount of radioactive material eluted as immune complex, and thereby followed the clearance of these complexes from the blood; there was no significant difference in the rate of clearance of immune complexes between mice bearing HX99 tumours (which gave a high localization index) and those bearing HX106 tumours (which gave no specific localization) (Fig. 5).

We observed that, in both tumour-bearing and tumour-free mice, the specific (anti-CEA) IgG was cleared more rapidly in the first hour after injection than the nonspecific Ig. The specific $^{125}$I-labelled IgG also contained a small amount of aggregated material (Fig. 4) which was absent from the $^{131}$I-labelled nonspecific Ig. It is possible that the anti-CEA antibodies were more susceptible to damage during iodination, and that this resulted in an initial difference in behaviour between the two types of IgG.

To ascertain whether immune complexes of CEA and anti-CEA might localize in tumours through their content of histiocytes, we took C57 BL mice carrying a murine fibrosarcoma (FS6) (Mantovani *et al.*, 1977), which has a macrophage population of 25%, and administered both

$^{125}$I-labelled anti-CEA with CEA at equivalence and $^{131}$I-labelled goat Ig. No specific localization in the tumour was detected.

### DISCUSSION

Other workers have successfully demonstrated the localization of specific anti-CEA antibodies in human colonic carcinoma xenografted either into hamsters (Primus *et al.*, 1973) or into nude mice (Mach *et al.*, 1974). We have expanded this work by showing that anti-CEA antibodies will localize in human breast carcinomas.

The quantity of CEA circulating in the plasma of these mice will depend on the rate of synthesis and release of CEA by the tumour, and also on its rate of clearance. Our results suggest that the degree of localization of specific antibodies is dependent on the amount of CEA stored in the tumour, and that the amount of circulating CEA is irrelevant. The latter observation accords with the clinical experience recorded by Goldenberg and his co-workers (Goldenberg *et al.*, 1978; Nagell *et al.*, 1980; Primus *et al.*, 1980). This is important since it indicates that plasma CEA levels cannot be used to select patients for the application of this technique. The amount of CEA in the tumour as estimated either by an immunohistochemical stain or by extraction and subsequent radioimmunoassay may be a more reliable guide.

The results accord with the hypothesis that the anti-CEA antibodies are localized in the tumour by reaction with CEA at that site. In relation to this, we have shown by autoradiography that monoclonal antibodies raised to cell-surface antigens of human teratoma xenografts localize in close association to viable tumour cells after *in vivo* administration of the antibodies (Moshakis *et al.*, 1981).

Many tumours contain large numbers of histiocytes, and it could be visualized that specific antibodies would be localized in tumours, owing to uptake of immune complexes by the histiocytes. Although we looked for evidence to support this alternate hypothesis we found none. The levels of circulating immune complexes were similar in mice bearing HX99 tumours and those bearing HX106, yet one tumour showed a high degree of localization of specific antibody, whilst the other had none. Furthermore, anti-CEA alone and anti-CEA/CEA complexes failed to localize in a murine fibrosarcoma which is known to have a high percentage of macrophages in its cell population.

Although in this system, using these particular tumours, the localization index increased with time, the total amount of radioactivity in the tumours decreased. The optimal time at which to use an external radioscan in patients is balanced between the two factors. The other point to note is that at all times, apart from the tumour, the most radioactive tissue was the blood. This emphasizes the need for a technique to subtract the activity in the blood pool.

Having established the usefulness of this model system in relation to the well-known antigen CEA, we are now in a position to extend this work to other less well studied antigens and to patients. Experiments have started using affinity-purified antibodies to the epithelial membrane antigen (EMA) expressed by human breast carcinomas (Heyderman *et al.*, 1979; Sloane & Ormerod, 1981) and to teratomas, through the use of monoclonal antibodies (Moshakis *et al.*, 1981). We hope that this latter approach can, in time, be extended to the study of human breast carcinomas.

We are grateful to Mrs K. Steele, Mrs S. Imrie and Mr C. Day, of the Institute of Cancer Research, for their skilful technical assistance.

Dr M. G. Ormerod and Dr J. H. Westwood were supported by project grants from the Medical Research Council.

### REFERENCES

Bailey, M. J., Gazet, J. C. & Peckham, M. J. (1980) Human breast carcinoma xenografts in immune suppressed mice. *Br. J. Cancer*, **42**, 524.

Bailey, M. J., Neville, A. M., Ormerod, M. G. & 4 others (1981) Comparative functional histopathology of human breast carcinoma xenografts. *Br. J. Cancer*, **43**, 125.

COOMBES, R. C., POWLES, T. J., GAZET, J. C., FORD, M. T., MCKINNA, A. & NAVILLE, A. M. (1980) An assessment of biochemical tests to screen for metastases in the follow-up of patients with breast cancer. *Lancet*, i, 296.

DYKES, P. W., HINE, K. R., BRADWELL, A. R. & 4 others (1980) Localisation of tumour deposits by external scanning after injection of radiolabelled anti-carcinoembryonic antigen. *Br. Med. J.*. **280**, 220.

GOLD, P. & FREEDMAN, S. O. (1965) Demonstration of tumour-specific antigens in human colonic carcinomata by immunological and adsorption techniques. *J. Exp. Med.*, **121**, 439.

GOLDENBERG, D. M., DELAND, F., KIM, E. & 6 others (1978) Use of radiolabelled antibodies to carcinoembryonic antigens for the detection and localisation of diverse cancers by external photoscanning. *N. Engl. J. Med.*, **298**, 1384.

GREENWOOD, F. G., HUNTER, W. M. & GLOVER, J. S. (1963) The preparation of $^{131}$I-labelled human growth hormone of high specific activity. *Biochem. J.*, **89**, 114.

HEYDERMAN, E. & NEVILLE, A. M. (1976) A shorter immunoperoxidase technique for the demonstration of carcinoembryonic antigen and other cell products. *J. Clin. Pathol.*, **30**, 138.

HEYDERMAN, E., STEELE, K. & ORMEROD, M. G. (1979) A new antigen on the epithelial membrane: Its immunoperoxidase localisation in normal and neoplastic tissue. *J. Clin. Pathol.*, **32**, 35.

LAURENCE, D. J. R., STEVENS, U., BETTELHEIM, R. & 6 others (1972) Role of plasma carcinoembryonic antigen in diagnosis of gastrointestinal, mammary and bronchial carcinoma. *Br. Med. J.*, iii, 605.

MACH, J.-P., CARREL, S., FORNI, M., RITSCHARD, J., DONATH, A. & ALBERTO, P. (1980) Tumour localisation of radiolabelled antibodies against carcinoembryonic antigen in patients with carcinoma. *N. Engl. J. Med.*, **303**, 5.

MACH, J. P., CARR, S., MERENDA, C., SORDAT, B. & CEROTTINI, J.-C. (1974) *In vivo* localisation of radiolabelled antibodies to carcinoembryonic antigen in human colon carcinoma grafted into nude mice. *Nature*, **248**, 704.

MANTOVANI, A., EVANS, R. & ALEXANDER, P. (1977) Nonspecific cytotoxicity of spleen cells in mice bearing transplanted chemically induced fibrosarcomas. *Br. J. Cancer*, **36**, 35.

MOSHAKIS, V., MCILHINNEY, R. A. J., RAGHAVAN, D. & NEVILLE, A. M. (1981) Monoclonal antibodies to detect human tumours: An experimental approach. *J. Clin. Pathol.*, **34**, 314.

NAGELL, J. R., KIM, E., CASPER, S. & 4 others (1980) Radioimmunodetection of primary and metastatic ovarian cancer using radiolabelled antibodies to carcinoembryonic antigen. *Cancer Res.*, **40**, 502.

ORMEROD, M. G. (1978) Antigenic determinants of carcinoembryonic antigen. *Scand. J. Immunol.*, **8**, (Suppl. 8), 433.

PRESSMAN, D. (1957) Radiolabelled antibodies. *Ann. N.Y. Acad. Sci.*, **69**, 644.

PRIMUS, F. J., BENNETT, S. J., KIM, E. E., DELAND, F. M., ZAHN, M. C. & GOLDENBERG, D. M. (1980) Circulating immune complexes in cancer patients receiving goat radiolocalising antibodies to carcinoembryonic antigen. *Cancer Res.*, **40**, 497.

PRIMUS, F. J., WANG, R. H., GOLDENBERG, D. M. & HANSEN, H. J. (1973) Localisation of human GW-39 tumours in hamsters by radiolabelled heterospecific antibody to carcinoembryonic antigen. *Cancer Res.*, **33**, 2977.

SLOANE, J. P. & ORMEROD, M. G. (1981) Distribution of epithelial membrane antigen in normal and neoplastic tissues and its value in diagnostic tumour pathology. *Cancer*, **47** (in press).

STEEL, G. G., COURTENAY, V. D. & ROSTOM, A. Y. (1978) Improved immunosuppression techniques for the xenografting of human tumours. *Br. J. Cancer*, **37**, 224.

TORMEY, D. C. & WAALKES, P. (1978) Clinical correlation between CEA and breast cancer. *Cancer*, **42**, 1507.

WESTWOOD, J. H. & THOMAS, P. (1975) Studies on the structure and immunological activity of carcinoembryonic antigen: The role of disulphide bonds. *Br. J. Cancer*, **32**, 708.

WESTWOOD, J. H. (1978) Carcinoembryonic antigen: Its chemistry. In *Tumour Markers Determination and Clinical Role*. Ed. Griffiths *et al.* Cardiff: Alpha Omega. p. 15.